# Differences in Executive Functioning in Children with Attention Deficit and Hyperactivity Disorder (ADHD)

**DOI:** 10.3389/fpsyg.2017.00976

**Published:** 2017-06-20

**Authors:** M. Rosa Elosúa, Sandra Del Olmo, María José Contreras

**Affiliations:** ^1^Psicología Básica I, Universidad Nacional de Educación a DistanciaMadrid, Spain; ^2^Hospital Clínico Universitario Lozano BlesaZaragoza, Spain

**Keywords:** executive functions, Attention Deficit and Hyperactivity Disorder, inhibition, divided attention, updating, attentional shifting

## Abstract

In recent years, the interest in Attention Deficit and Hyperactivity Disorder (ADHD) and its relation to deficits in working memory (WM) and more specifically the different executive functions (EFs) has grown, to the point of confirming that these are quite frequent in this disorder. The aim of this study was precisely to explore differences in executive functioning of WM in fourth grade Primary school children with and without ADHD (26 and 29 children, respectively), introducing rigorous control measures in the tests used. Four EFs were analyzed: divided attention, updating, attentional shifting and inhibition, measured through four tasks, the dual-task paradigm (digits and box-crossing), the N-Back task, the Trail Making Test and the Stroop task, respectively. The results showed that participants with ADHD, compared to children with typical development (TD), exhibited a smaller verbal memory span as well as deficits in the attentional shifting and updating functions. However, a similar performance for the EF of inhibition was found for both groups of participants. Finally, an unexpected result was obtained with regard to the role of divided attention, as children with ADHD were less impaired when performing the double task than participants in the TD group.

## Introduction

The Attention Deficit and Hyperactivity Disorder (ADHD) is a neurodevelopmental disorder characterized by a pattern of inattention and/or hyperactivity-impulsivity above that expected for the individual’s level of development. It affects daily life in a clinically significant way and it is present in multiple contexts, hindering academic and work performance, as well as social development. Inattention, hyperactivity, and impulsivity can manifest at a behavioral and cognitive level in different ways, so that it is observed that children with ADHD are often distracted, they have difficulty in sustaining attention over prolonged periods of time, they get up frequently and inappropriately within the situation in which they find themselves, they struggle to remain still, they disrupt the activities of others or respond without thinking and in a disorganized way. A minimum number of these clinical symptoms must be present before the child is 12 years old. The disorder can present itself in a mild, moderate or severe level, and the severity of the symptoms may vary across time (DSM-5; American Psychiatric Association, 2013).

Attention Deficit and Hyperactivity Disorder has been considered one of the most common disorders in childhood, with a prevalence of approximately 3 to 5% (Willcutt, 2012). It has a greater occurrence in boys than in girls, with a ratio of three boys for every girl (Ramtekkar et al., 2010) and the combined subtype is the most frequent subcategory. In many cases, these children do not see a specialist until third or fourth grade of Primary school, when their academic performance is unsatisfactory, possibly due to the increasing demands in school tasks in comparison with previous years. Their ability to concentrate and to organize becomes insufficient to achieve adequate learning. Therefore, it is possible that their academic performance is lower than expected given their IQ. Moreover, their social functioning and emotional well-being is also affected, with its subsequent impact on their families (Mannuzza et al., 2004).

Among the difficulties faced by children with ADHD, executive functions (EFs, hereinafter) have often been pointed out (Willcutt et al., 2005; Lambek et al., 2011; Re et al., 2015). The EFs is a challenging topic to study, as not only is it elusive to define, but is also difficult to measure (see Jurado and Roselli, 2007; Miyake and Friedman, 2012). The EFs refer to the set of skills that allow the generation, supervision, regulation, and implementation of behaviors appropriate to achieve complex goals, especially those that are not automated and that allow us to address new situations (Miyake and Friedman, 2012). Any alteration in the development of these functions would be reflected in difficulties in making decisions, respecting rules, regulating emotions, having successful social relationships, or ensuring new learning. Moreover, these EFs could be considered as part of the attentional control of working memory (WM), which has a great influence on academic performance (Dehn, 2008; McCloskey et al., 2009; Langberg et al., 2013; García-Madruga et al., 2014; Miranda et al., 2015).

As to the neural basis of EFs seem to be localized significantly in the prefrontal cortex (Fuster, 2008). Some studies have reported that alterations in prefrontal areas produce neuropsychological deficits, particularly in EFs, as well as behaviors of impulsivity, hyperactivity, and inattention. Hence, this area has also been linked to ADHD (e.g., Geurts et al., 2004). These alterations have been found both, in childhood/adolescence and in adulthood, which may indicate they are of stable nature (Biederman et al., 2007).

In the extensive meta-analysis carried out by Willcutt et al. (2005), the authors concluded that ADHD is associated with deficits in WM and various EFs, with inhibition and planning being particularly deteriorated. For this reason, and based on the accumulated evidence suggesting that deficits in EFs have a high prevalence in ADHD, different authors have proposed that the EF performance is the cognitive mechanism that best differentiates participants with and without ADHD (e.g., Boonstra et al., 2005), renaming ADHD as an EF disorder.

Nevertheless, although most studies report deficient results in tasks where EFs are involved, not all children with ADHD invariably exhibit deficits in such functions (Nigg et al., 2005; Lambek et al., 2010; Duff and Sulla, 2015). Nigg et al. (2005) observed that almost 80% of children with ADHD exhibited a deficit in at least one EF, while this only occurred in 50% of children with typical development (TD). Furthermore, a greater number of deficits were present in the ADHD group for the different EFs. Similar results were obtained by Lambek et al. (2010), which entails that although a significantly greater impairment of the EFs is present among the ADHD group, this does not occur in every case or for the same EFs. For this reason, although EFs seem to be affected in this disorder, such affectation is heterogeneous (Sonuga-Barke, 2005), creating the need to deepen the study of the different EFs and their possible and varied performance patterns in ADHD.

In this study, the three main EFs used by Miyake et al. (2000) were chosen (attentional shifting, updating, and inhibition). These three functions were chosen because as Miyake et al. (2000) said “they seem to be relatively circumscribed, lower level functions (in comparison to some other often postulated EFs like “planning”) and hence can be operationally defined in a fairly precise manner” (p. 55). Divided attention was added, as one of the most important EFs named and used by Baddeley (2002). Hence, while some studies have examined some of these functions and other studies have worked with other functions, a novel aspect of the present study is precisely the joint use of these four EFs, for the reasons mentioned above. Below, we review these four EFs (divided attention, attentional shifting, updating, and inhibition) and briefly discuss some previous studies carried out with the tasks we have chosen as measures of each EF. Details of each task are provided in the “Materials and Methods” section.

One of the tasks most commonly used to measure divided attention is the dual-task paradigm. The present study uses the dual task paradigm proposed by Baddeley et al. (1997), as it allows for the level of memory span of each participant to be adapted during the performance of the task. Participants must carry out each of the tasks separately first to subsequently carry them out together. In the latter condition, a worse performance is expected, understanding that the secondary task causes interference with the primary task, competing for the same WM (hereinafter) resources. Studies on divided attention with ADHD evaluated through other dual task paradigms have yielded contradictory results. On the one hand, some studies have shown that, in children with ADHD, deficits appear in the divided attention function (Karatekin, 2004; Fuggetta, 2006). For example, Fuggetta (2006) used a dual task that requires the coordination of two task responses, a shift task that makes it necessary to disengage attention from one task and engage onto another, and a stimulus–response spatial compatibility task that requires participants to inhibit a prepotent response. Results indicated that the ADHD group (9–11 years old) needed significantly more time than the TD group to coordinate both responses from the dual task, to disengage their attention from one task to another and to inhibit a prepotent response.

On the other hand, other studies have reported no differences between the ADHD and TD groups, or between the disorder’s different subtypes (Inasaridze and Bzhalava, 2010). The present study involved a sample of ADHD children aged between 6 and 16 years and found that the increase of difficulty with the dual task (a list memory task and a computerized tracking task or paper and pencil motor tracking task) did not disproportionately affect children and adolescents with ADHD with respect to the TD group.

The Trail Making Test (TMT) has been traditionally used to evaluate attentional shifting, especially part B of the test (Pennington and Ozonoff, 1996). It has been reported that the time taken to perform the TMT-B can discriminate between participants with and without ADHD (see Pennington and Ozonoff, 1996; Willcutt et al., 2005). Time and errors committed in this test may be due to the impulsive responses that people with ADHD have when presented with stimuli to which they must respond to sequentially. There is repeated evidence that they take longer and make more mistakes than those without ADHD (Wodka et al., 2008; Hale et al., 2009). Nevertheless, it has occasionally been found that this test does not have an adequate predictive power to differentiate between them (Perugini et al., 2000).

The measurement of the updating function may be addressed through the N-Back task. Although there have been few studies published that used this task on children with ADHD, deficits have been found that yield greater difficulties as the workload of the test increases (Shallice et al., 2002; Karatekin et al., 2009; Bechtel et al., 2012). In Shallice et al.’s (2002) study, differences were found in the N-Back task for both groups of children with ADHD (7- to 8-year-old and 9- to 12-year-old), compared to their age-matched TD groups, obtaining worse results for the ADHD group. Regarding the number of hits, a significant effect of both group and age was found, as well as for the N-Back condition. However, the interaction effect was not significant. Therefore, ADHD children yielded less hits than TD children; Moreover, 7- to 8-year-old participants obtained more errors than the 9- to 12-year-old group. Finally, both groups obtained a significantly greater number of hits in the 0-back condition than in the 1-back condition, and more in the latter than in the 2-back condition.

One of the most important paradigms used to evaluate the role of inhibition has been the Stroop Test. Some authors have considered inhibitory control as the key component in ADHD, referring to it as the very essence of the disorder (Barkley, 1997). There have been many publications that have found that there is a greater interference in the Stroop test in children with ADHD compared to those without this disorder (Lansbergen et al., 2007; Wodka et al., 2008). However, some authors have argued that, rather than an interference effect, what is most often seen in ADHD is a slower response time, a reduced accuracy and/or greater variability, in comparison to TD group (Nigg et al., 2002; van Mourik et al., 2005; Schwartz and Verhaeghen, 2008). For this reason, the validity of the Stroop test to evaluate the inhibitory behavior has been questioned and criticized for evaluating other neurocognitive functions other than interference control (van Mourik et al., 2005).

Given the discrepancy of previous studies regarding the deterioration in EF performance in ADHD, the novelty of this study will apply the necessary control measures in the tests used to avoid the mistakes derived from the clinical approach, which although it uses tests of experimental tradition, it does not always have the rigor and control of this methodology, leading to contradictory results (see Snyder et al., 2015). Therefore, this present study takes into account both traditions, the clinical approach (as data are collected in a hospital and we are interested in the clinical application that its results may entail), and also all the necessary controls to correct all questions that have arisen in the revised bibliography regarding the tests used.

In this context, the main objective of the study was to assess the performance of EFs and WM, according to Baddeley and Hitch (1974); Baddeley, 2012) model, in children with ADHD compared to children with TD. To do this, the following EFs were assessed: divided attention, attentional shifting, updating, and inhibition. Our initial hypotheses pose that if any of these functions were affected in ADHD participants, a significantly lower performance would be obtained in the tasks used to examine these EFs, in comparison to TD children.

## Materials and Methods

### Participants

The study included 26 children with ADHD (20 with ADHD combined subtype and 6 with ADHD inattentive subtype) who visited for the first time the Child and Adolescent Psychiatry outpatient unit of a General Hospital. Children with ADHD had a previous clinical diagnosis of ADHD that was confirmed before their participation in the study. They were not taking medication. The inclusion criteria to participate in the group of children diagnosed with ADHD were: (a) fulfilling the diagnostic criteria for Attention Deficit and Hyperactivity Disorder (DSM-IV-TR; American Psychiatric Association, 2000) as a main diagnosis, and without any other known diagnosis, although emotional or behavioral symptomatology may be comorbidly present; (b) being aged between 9 and 10 years of age and/or currently enrolled in fourth grade of Primary school; (c) not have been under any psychological and/or drug treatment for ADHD before; (d) having an IQ equal to or above 85. Furthermore, a group of 29 children with TD and similar sociodemographic characteristics to the ADHD group were included in the study. All children in TD group were students from fourth grade of Primary from a public school, as that age group is considered critical in relation to the behavioral problems that arise. The inclusion criteria for TD group were the same as for ADHD group, with the exception of the ADHD diagnosis. **Table 1** shows the sociodemographic and clinical characteristics of the sample used in this study.

**Table 1:** Clinical and sociodemographical characteristics of the sample with means and proportions (standard deviation within parentheses).

	ADHD children (*n* = 26)	TD children (*n* = 29)
Age	9.24 (0.40)	9.18 (0.43)
Sex (male/female)	17/9	13/16
IQ	95.15 (1.98)	97.17 (1.70)
Repeats school year	11	5
Nationality (Spanish/Foreign)	23/26	23/29
Parents civil status (married/separated)	21/5	24/5
Parents’ education (%)		
- Father		
Primary	23	38
Secondary	39	31
High school	8	3
Professional apprenticeships	15	17
University studies	15	11
- Mother		
Primary	31	38
Secondary	27	31
High school	12	7
Professional apprenticeships	4	7
University studies	26	17
Parents’ profession (%)		
- Father		
Qualified professional	12	10
Technical personnel	38	24
Non-technical personnel	50	65
- Mother		
Qualified professional	8	7
Technical personnel	27	24
Non-technical personnel	65	69

### Materials

#### Clinical Assessment

The clinical interview collected the symptomatology presented by children with ADHD, as well as the sociodemographic data of all participants. Additionally, the Attention Deficit and Hyperactivity Disorder Assessment Scale (EDAH in Spanish) by Farré and Narbona (2013) was also administered to the ADHD group, which evaluated the symptoms associated with ADHD. In order to fulfill this rating scale, the information collected by these children’s teachers on their usual behavior in the school context was included. This scale was validated in Spain on children and scores were recorded in three scales: Hyperactivity, Attention Deficit, and Behavior Disorder.

#### Neuropsychological Tasks

##### K-BIT: Kaufman Brief Intelligence Test (Kaufman and Kaufman, 2000)

This test consists of a vocabulary subtest, influenced by school related skills, that measures verbal skills, and a matrix subtest that assesses non-verbal skills through the perception of relationships and the completion of visual analogies. The score used was the total IQ, obtained using both subtests.

##### Digit Span Task

The participant is presented with a series of digits orally that he/she has to recall immediately thereafter, in the same order in the first part of the task and in reverse order in the second part of the task. The length of the trials is progressively increased at each level of amplitude; starting with series of two digits and adding one digit with each level. Each level has nine trials of the same length, divided into series of three. A series is successfully achieved when the participant does not make any errors in at least two of the three trials. If the participant does not achieve at least two of the three series within each level, the task is ended, scoring the span level of the last level that the participant achieved successfully, that is, the last level in which he/she completed at least two series successfully. In this task, one point is awarded for each span level successfully achieved.

##### Dual-Task Paradigm (Baddeley et al., 1997)

To examine divided attention we used the dual-task paradigm, which involves a digit recall task, adapted to the span level of each participant’s WM, and a box-crossing task, where the participant crosses boxes following a path laid out on a sheet with 80 boxes as quickly as possible during 2 min (paper and pencil task). After this time, the task is terminated. First, tasks are performed separately (single task) and afterward, combined (dual task), lasting 2 min each. The *mu* index of the dual task is calculated to measure the distribution of attention as follows:

*μ* = [1 – (*p_m_* + *p_t_*)/2] × 100,

where *p*_m_ is the proportion of digit correct series in the single task subtracting the digit correct series from the dual task and *p*_t_ is the number of crossings in the single task minus those in the dual task and then divided by the number of crossings made during the single task. With this formula the performance percentage of the subject in the double task with respect to the single task (Baddeley et al., 1997) is obtained through the *mu* index. The main dependent variable was the distribution of attention measured by *mu* index. The proportion of digit correct series in single and dual tasks were also dependent variables recorded as well as the number of box-crossing put in single and dual tasks. Test–retest reliability is variable according to some studies (between 0.44 and 0.67).

##### Trail Making Test (Reitan and Wolfson, 1993)

We used the TMT to assess the EF of attentional shifting. This test consists of two parts A and B. In Part A, the participant must link numbers from 1 to 25 in ascending order as quickly as possible without lifting the pencil from the paper. In Part B, participants must link numbers from 1 to 13 in ascending order and the letters A to L in alphabetical order, alternating numbers and letters: 1-A-2-B-3-C, etc. The examiner asks the participant to correct his/her own mistakes, with the increase in time that this entails. To minimize the effects of motor speed and visual tracking speed in the execution of this test, the B-A and B/A indexes are obtained as dependent variables (Arbuthnott and Frank, 2000), plus the time in seconds that they take to complete each of the parts of the test. Test–retest reliability is variable according to some studies (between 0.60 and 0.90).

##### N-Back Task (Braver et al., 1997)

To examine the EF of updating, the N-Back paradigm with three conditions (1-back, 2-back, and 3-back) was used. In each of these, 20 letters are presented at the rate of one per second. In the 1-back condition, the participant has to indicate when he/she detects the same letter twice in a row; in the 2-back condition, when the same letter is repeated but separated by a single different letter; and in the 3-back condition, he/she must indicate when he/she detects the same letter separated by two different letters. The dependent variable was the total number of errors recorded, specifying whether they are errors of omission, false alarm or perseveration. Test–retest reliability is variable according to some studies (between 0.65 and 0.92).

##### Stroop Test (Golden, 1978)

The Stroop test was used to examine the EF of inhibition. The test consists of three sheets of 100 elements each: the Word condition (W), the Color (C) condition and, finally, the Word-Color (WC) condition. In the three conditions, participants have to read aloud all the elements that they can in 45 s. The number of elements obtained in the three conditions and the interference index, which is calculated from the results of the other conditions [WC - (W × C/W + C)], are analyzed as dependent variables. Test–retest reliability is variable according to some studies (between 0.71 and 0.98).

### Procedure

The Ethics committees of the University (UNED) and the General Hospital approved the study, in accordance with the Declaration of Helsinki. Oral permission from the children and written informed consent from their parents were obtained before beginning the evaluation. In the case of the participants with ADHD, the tests were applied in the Child and Adolescent Psychiatry outpatient unit at the General Hospital. The assessment was performed by a doctoral-level clinical psychologist in a single session, which lasted an hour and a half, approximately. In the TD group, the application of the tests lasted approximately 1 h. For both groups, the order of administration of the tests was counterbalanced, except for the clinical interview and the K-BIT, which were always applied at the beginning of the session. Halfway through the application of the tests, a rest period was granted to avoid fatigue in the participants.

## Results

The statistical analyses of the results were performed using parametric tests. In order to compare the sociodemographic characteristics between groups, the Student *t*-test was used (verifying the homogeneity of variances with Levene’s test) for the quantitative variables (age and IQ) and the chi-square test was used to compare qualitative variables (sex, repetition of school year and the parents’ sociocultural characteristics). No significant differences between groups in the sociodemographic variables were found, except for the repetition of the school year variable, which showed a greater number of repetitions for the ADHD group (*z* = 4.18; *p* = 0.04) than for the TD group. As for the EDAH scores for the ADHD participants, all were above the 80th percentile regarding the presence of clinical symptoms of hyperactivity, attention deficit, and behavioral disorders. **Table 2** presents the main results of the study.

**Table 2:** Means (and standard deviations within parentheses) for the dependent variables used (minimum and maximum values) in the four tasks performed by ADHD and TD children, Student *t*-test and Cohen’s *d*.

	ADHD children (*n* = 26)	TD children (*n* = 29)	Students *t* (significance)	Cohen’s *d*
Direct digits (min 3–max 6)	4.27 (0.67)	4.83 (0.66)	-3.12 (0.01)	0.84
Reversed digits (min 2–max 5)	3.08 (0.89)	3.66 (0.61)	-2.83 (0.01)	0.76
Total digits (min 5–max 11)	7.35 (1.38)	8.48 (1.12)	-3.36 (0.01)	0.90
Digits single task (min 0.13–max 1)	0.69 (0.19)	0.70 (0.15)	-1.12 (0.91)	0.06
Digits dual task (min 0.18–max 1)	0.72 (0.21)	0.68 (0.17)	0.72 (0.48)	-0.21
Box-crossing single task (min 45–max 146)	89.77 (23.60)	118.79 (14.95)	-5.51 (0.01)	0.47
Box-crossing dual task (min 39–max 156)	89.08 (27.35)	102.38 (29.32)	-1.73 (0.09)	0.47
*Mu* index (min 61.79–max 128.02)	101.16 (13.04)	91.84 (12.60)	2.70 (0.01)	-0.73
TMT-A (s) (min 27–max 105)	62.35 (15.79)	57.52 (17.68)	1.06 (0.29)	-0.29
TMT-B (s) (min 80–max 503)	195.15 (100.01)	144 (52.07)	2.42 (0.02)	-0.64
B/A index (min 0.7–max 6.28)	3.12 (1.10)	2.51 (1.13)	2.04 (0.05)	-0.55
B-A index (min–max)	132.81 (90.92)	86.48 (48.66)	2.39 (0.02)	-0.64
Stroop-Word (min 65–max 143)	99.15 (16.72)	117.34 (10.14)	-4.94 (0.01)	1.32
Stroop-Color (min 55–max 96)	72.00 (8.93)	76.07 (8.65)	-1.72 (0.09)	0.46
Stroop Word-Color (min 32–max 59)	43.00 (5.73)	45.79 (5.68)	-1.81 (0.08)	0.49
Stroop Interference (min -25.48–max 37.24)	1.68 (11.03)	-0.28 (4.83)	0.87 (0.39)	-0.23
1-Back (errors) (min 0–max 10)	1.85 (2.43)	1.00 (1.07)	1.7 (0.09)	-0.45
2-Back (errors) (min 0–max 10)	5.04 (2.54)	3.10 (2.06)	3.12 (0.01)	-0.84
3-Back (errors) (min 1–max 11)	6.35 (2.15)	5.62 (1.47)	1.47 (0.15)	-0.40
N-Back (total errors) (min 2–max 27)	13.23 (5.81)	9.72 (3.27)	2.78 (0.01)	-0.80
N-Back (omissions) (min 2–max 19)	8.96 (4.37)	7.48 (2.42)	1.58 (0.12)	-0.42
N-Back (false alarms) (min 0–max 21)	2.27 (4.28)	0.83 (1.04)	1.76 (0.08)	-0.46
N-Back (perseverations) (min 0–max 4)	2 (1.27)	1.41 (1.18)	1.78 (0.81)	-0.48

### Digit Span Level

The results indicated that the ADHD group had a lower score than the TD group, both in direct order digits (*t* = -3.12, *p* < 0.01) and in the reverse order (*t* = 2.83, *p* = 0.01).

### Dual-Task Paradigm

In the digit recall task, no significant differences between groups were obtained either for the single task (*t* = -1.12; *p* = 0.91) or for the double task (*t* = 0.72; *p* = 0.48). Regarding the box-crossing task, no significant differences between groups were obtained for the dual task (*t* = -1.73; *p* = 0.09); however, significant differences were found for the single task (*t* = -5.51; *p* = 0.01). Participants with ADHD obtained worse scores than TD (see **Table 2**).

The results for the *mu* index showed that there were significant differences between groups (*t* = 2.70; *p* = 0.01), but not in the expected direction, considering our initial hypothesis. That is, the ADHD group outperformed the TD group in the distribution of attention.

Finally, it was also analyzed whether there were differences within each of the two groups when they performed the single and the dual task. In the ADHD group, similar results were obtained in the performance of the digits task (*t* = -0.65; *p* = 0.52) and in the box-crossing task (*t* = 0.21; *p* = 0.84) when they performed them as a single task and in the dual task mode. For the TD group, there were no differences between the single and dual tasks (*t* = 0.63; *p* = 0.53), but in the case of the box-crossing task, the TD group lowered their performance in the dual task (*t* = 3.78; *p* < 0.01).

### Trail Making Test

The results indicated that the ADHD group used more time than the TD group to complete the Part B of TMT (*t* = 2.42; *p* = 0.02), yet there were no differences between groups in Part A (*t* = 1.06; *p* = 0.29). Moreover, there were significant differences between groups for both, the B-A index (*t* = 2.39; *p* = 0.02) and the B/A index (*t* = 2.04; *p* < 0.05). It shows that the ratio of the time spent performing Part B with respect to Part A of the TMT differs between the two groups, with participants with ADHD needing significantly more time to perform Part B (more complex) than to perform Part A.

### N-Back

A 2(group) × 3(condition: 1-back, 2-back, and 3-back) repeated measures ANOVA was performed for the second factor, considering the total number of errors as the dependent measure. The results indicated that the main effect of the N-Back condition was significant [*F*(1,53) = 119.57, *MC* = 295.40, *p* < 0.001, $\eta \frac{2}{\text{p}}$ = 0.69]. The fit of the Bonferroni multiple comparisons indicated that all comparisons between levels of *n*-back were significant (*p* = 0.001). Therefore, 1-back obtained a significantly lower mean of errors than 2-back and 3-back; 2-back obtained a significantly greater mean of errors than 1-back and lower than 3-back; and 3-back obtained significantly more errors than 1-back and 2-back. Similarly, the group factor was significant [*F*(1,53) = 7.82, *MC* = 56.20, *p* = 0.007, $\eta \frac{2}{\text{p}}$ = 0.13]. The Bonferroni multiple comparisons indicated a greater mean of errors for the ADHD group than the TD group (*p* = 0.007). However, only a trend toward significance was observed in relation to the interaction of the variables (*p* = 0.08). *A posteriori* analyses using the Bonferroni method were conducted to determine between which N-Back condition the differences occurred. Pairwise comparisons applying the Bonferroni correction indicated that the condition that best distinguished performance between the two groups was only the 2-back (*p* < 0.05). ADHD group had more significant errors than TD group. Finally, no significant differences between groups were found for the type of error performed [omission (*p* = 0.12); false alarm (*p* = 0.08); perseveration (*p* = 0.81)], although the trend toward significance of the false alarms is remarkable.

### Stroop Test

The results showed significant differences between groups in the Stroop-Word task (*t* = -4.94; *p* = 0.01), with the performance of the ADHD group obtaining worse scores than the TD. However, there were no differences between groups with respect to the Stroop-Color (*t* = -1.72; *p* = 0.09), Stroop Word-Color (*t* = -1.81; *p* = 0.08) or the Stroop Interference (*t* = 0.87; *p* = 0.39), although, in the first two conditions, clear trends toward significance were obtained.

To analyze the relationships between the four EFs, the correlations between them were obtained, but only a statistically significant negative correlation was found between the errors obtained in the N-Back and the resistance to interference in the Stroop test (*r* = -0.29, *p* < 0.05). It is important to note the limitations of these results, due to the fact that two extreme groups (ADHD and TD) were considered together. **Table 3** gathers these results.

**Table 3:** Bivariate correlations among executive functions and the verbal span and IQ tasks.

Bivariate correlations (*n* = 55)	K-Bit	Direct digits	Reversed digits	Stroop Interference	Stroop Word	TMT-B	N-Back errors
Direct digits	0.37^∗∗^						
Reversed digits	0.45^∗∗^	0.62^∗∗^					
Stroop Interference	0.05	0.01	-0.06				
Stroop Word	0.31^∗^	0.55^∗∗^	0.53^∗∗^	-0.27			
TMT-B	0.41^∗∗^	-0.18	-0.32^∗^	0.12	-0.36^∗∗^		
N-Back (errors)	-0.49^∗∗^	-0.44^∗∗^	-0.42^∗∗^	-0.29^∗^	-0.35^∗∗^	0.26	
*Mu*	-0.20	-0.07	-0.04	0.08	-0.10	0.06	0.12

*^∗^p < 0.05, ^∗∗^p < 0.01.*

## Discussion

The main objective of this study was to assess the functioning of WM and EFs in a sample of fourth grade Primary school children with ADHD, compared to a TD group. In general, most of the examined functions were affected in the cognitive functioning of children with ADHD, with the exception of the ability to attend to two things at once and the interference, in which no deficits were found in comparison to the TD group.

Regarding the Digit Span task, both groups had more difficulty in performing the digit task in reverse order than in direct order, and the span level obtained for the ADHD group was significantly lower than that for the TD group, both in the direct order and in the reverse order tasks. However, the results obtained in most of the previous studies report that children with ADHD have worse outcomes than TD in the reverse order of the Digit span task, but not in the direct order of this task (e.g., McInnes et al., 2003). It is possible that the rigorous control performed in our study, through the different levels of three series of digits per level, as well as the criteria to pass each level, have made the assessment of this task more sensitive, making it possible to objectify the differences in the performance of the groups. Thus, in ADHD, we have observed difficulties in both, the maintenance and handling of information. These deficits are related to the phonological loop and the central executive system, respectively.

Regarding the divided attention, measured through the performance in the dual-task paradigm (digits and box-crossing), significant differences were found between groups but in the opposite direction than what was expected. The percentage of distribution of attention (*mu*) for the dual task was higher in children with ADHD than TD, not only being less affected by the second task, but actually benefiting from it. In Inasaridze and Bzhalava (2010) study, which used a similar dual task paradigm (a list memory task and a computerized tracking task or paper and pencil motor tracking task), but without adjusting the level of verbal span of each participant, no differences were found. That is, the performance in the distribution of attention among children aged between 6 and 16 years with and without ADHD was similar. It is noteworthy that although this prior study used a greater age range and the conditions of the task differed (the contents of the memory list was not detailed and the span level of each participant was not adjusted), the attentional distribution index was very similar (*mu* index). Note that in our study, the digit span level for each participant was calculated in order to avoid differences in the performance of this paradigm due to the individual’s ability to perform the task separately. Therefore, the possibility that a poorer performance in the dual task was due to any reason other than the difficulty of distributing the resources between the two cognitive tasks was discarded. As Inasaridze and Bzhalava (2010) pointed out, in many of the studies that reported differences between groups with this type of task, the level of difficulty of the tasks performed separately has not been adapted to the levels of the individual ability of each participant. Only some studies have adapted the procedure to the level of each participant in the single digits task (e.g., Karatekin, 2004), similarly to ours. This is a good example of how our study has addressed the need for experimental control claimed by Snyder et al. (2015) in this type of research. The results of the present study, controlling for the memory span level of each participant and using a more homogeneous age group, are in line with those of Inasaridze and Bzhalava (2010), confirming that ADHD children’s divided attention, measured through this dual task paradigm, is not significantly affected in relation to that of the TD group.

As the single digits task, in our sample, we can confirm that there were no differences between groups and that neither group was affected by the addition of a second task. Moreover, in the case of participants with ADHD, even though it did not reach statistical significance, there was a slight improvement in their performance when they-performed the task together with the box-crossing task (while performance was maintained for the latter). Again, we found a result contrary to those obtained in previous studies in which a secondary task decreases the performance of the primary task. This paradoxical result may suggest that other psychological processes are coming into play, which is not controlled by our study. However, we would like to assess the plausibility of several explanatory arguments. It is possible that motivational factors may have influenced the performance of participants with ADHD in this task. As the cognitive-energetic model suggests, which emphasizes the role of factors such as effort, arousal, and activation in this disorder, the poor response execution in these children may be reflecting a non-optimal energy state (Sergeant, 2005). This would be consistent with ADHD motivational theories in terms that there is a greater aversion to low levels of stimulation. Accordingly, in our study, it is possible that participants in ADHD group were not affected by the dual task as they had a higher level of stimulation that could be optimal for them.

Other studies have obtained similar paradoxical results for children with ADHD in comparison to a TD group. For example, Grodzinsky and Barkley (1999) indicated that, in a digit recall task, there were only differences between groups in the direct order condition. As we have mentioned previously, this finding is contrary to what has been reported by other studies, where the deterioration in the reverse order digits task is more pronounced in the case of people with ADHD. When the task is better performed in reverse order of digits than in the direct order, it is plausible that this does not reflect a lack of ability, but rather, a lack of effort in the single task. Thus, issues such as the intrinsic interest in the task can improve performance.

Regarding the box-crossing task, a worse performance was obtained for the ADHD group, independently of whether it was performed alone or together with the digits task. The presence of differences between the TD group and the ADHD group in the box-crossing task when performed in single mode makes it impossible to confirm with certainty that its combination with other tasks can measure EFs. Therefore, it is possible that the difficulty of the secondary task in the dual-task paradigm is influencing these results, as it has proved to be an important feature. Karatekin (2004) found that participants with ADHD only had difficulty dividing their attention when the secondary task was cognitively demanding, hence, perhaps in our study our secondary task was not sufficiently complex and therefore did not achieve the expected effect.

Regarding the attentional shifting, our results indicated that the ADHD group needed more time to complete Part B of the TMT than the TD group and this result consistent with previous studies and meta-analysis (Pennington and Ozonoff, 1996; Willcutt et al., 2005; Wodka et al., 2008; Hale et al., 2009). However, there were no significant differences in the performance of Part A of the TMT. It is important to highlight that Part B of the TMT is the part related to cognitive flexibility and attentional shifting (Pennington and Ozonoff, 1996).

As Rohlf et al. (2012) indicated, the poorer performance in the TMT-B by participants with ADHD, compared to the TD group, could be explained to a certain extent by a lower performance in the TMT-A (which is not a measure of attentional shifting). Therefore, it not is advisable to use only the results of the TMT-B to assess performance in attentional shifting. It would be necessary to monitor the results in the TMT-A so that the difference between groups is not overestimated. In our case, both, the B/A index and the B-A index showed significant differences between groups, suggesting that the deficits observed in attentional shifting are not due to a slowing of cognitive and/or motor functions, but rather to a deficit in function of attentional shifting specifically. Therefore, for our sample, the hypothesis of the existence of deficits in the function of attentional shifting in participants diagnosed with ADHD was confirmed.

When interpreting the poorer performance in the TMT -such as deficits in attentional shifting-, it is important to note that the TMT-B has also been considered a measure of WM by certain authors (Boonstra et al., 2005). This might be reasonable, as the TMT-B requires keeping the last number or letter in mind while also looking for the next stimulus. The analyses performed in this study (see **Table 3**) showed correlations between the scores of the level of verbal span memory (digits in reverse order) with the TMT-B scores. Thus, our results could indicate that the performance in the TMT is relatively dependent on span memory, supporting Boonstra et al.’s (2005) hypothesis.

Regarding the EF of updating, measured through the N-Back task, it was observed that there was a deficient performance for the ADHD group, compared to the TD group. Overall, the ADHD participants made a significantly higher number of errors than the TD. Moreover, in both groups, the increased cognitive load of the task resulted in a greater number of errors during its execution. Thus, in the 3-back condition there were more errors than in the 2-back, and in the latter, more errors than in the 1-back, as has been reported previously in other studies (Shallice et al., 2002; Karatekin et al., 2009). The task condition that best discriminated between groups was the 2-back condition, thus being the optimum level of difficulty to study the impairment of this function, as it was not too easy (1-back) or too difficult (3-back), engaging the performance of participants with ADHD without greatly affecting the performance of the TD (see Pelegrina et al., 2015). In line with our study, Bechtel et al. (2012) obtained significant differences between groups only for the 2-back condition; especially highlighting that the easier condition of the task was not sensitive enough to distinguish their updating ability. Similarly, Ehlis et al. (2008) found that participants with ADHD had less activation in the ventro-lateral prefrontal cortex, especially during the 2-back condition, compared to the TD. It is noteworthy that the interaction effect (group × N-Back condition) was not significant in our study. We observed that the increment of the cognitive load within the different conditions of the task worsened the performance of both groups, without a significantly greater number of errors being made by ADHD participants when the difficulty of the task increased, although there was a significant trend. These same results were found in previous studies with children (Shallice et al., 2002) and young adults (Roberts et al., 2012).

As for the type of errors (omissions, false alarms or perseverations) made in the task, no significant differences were found between the two groups. There was, however, a trend toward significance in the case of false alarms, with participants with ADHD making a greater amount of this type of errors. Perhaps this result may be explained by the greater impulsivity that people with this disorder have, making it difficult for them to delay their answer and think about it before submitting it.

In our study, no deficits were observed in ADHD group regarding the interference condition of the Stroop task. This result is consistent with previous studies on children (Nigg et al., 2002). These data are important because, with the discrepancy of previous results in this task, our results show that differences only appeared between groups in the word reading condition, indicating that participants with ADHD were slower than those in the TD group. Similarly, although without finding significant differences between groups, it was observed that participants with ADHD also tended to be slower in the Color and Color-Word conditions. Therefore, these results support previous studies where no differences were found regarding interference, but also show a slower performance for other conditions (Nigg et al., 2002; van Mourik et al., 2005; Schwartz and Verhaeghen, 2008). It is worth highlighting some methodological aspects of the studies that have used this task previously and that could explain the appearance of contradictory findings in previous literature. Traditionally, when comparing participants with ADHD and TD of the same age, some researchers only analyzed their performance in the Color-Word Stroop test condition. The finding that participants with ADHD were slower in this condition was taken as evidence that they had more problems with response inhibition than the TD, regardless of the differences in reading speed of the groups (van Mourik et al., 2005). This is important, because as has been noted above, it has been reported that people with ADHD have lower scores for both, reading ability and color naming. Therefore, an interference score calculated exclusively from the Color-Word test would only be valid if there had been an adequate control of the speed differences in the rest of the conditions of the test, in order to avoid overestimating it. Once again, this highlights the need to compare results amongst studies that use the same conditions of the task and the same scoring indexes, as otherwise conflicting results could be explained by the different application conditions of the task. Similarly, some authors state that other cognitive processes may be intervening in the interference effect in addition to the inhibition function that may explain the results (MacLeod, 1991; cited in Sergeant et al., 2002). Hence, the importance of evaluating the specificity of the task to measure a cognitive process, or in its case, to perform a control over the intervening variables, is noteworthy.

As for the specificity of the four EFs studied, and limiting ourselves to the fact that the correlations obtained in our sample were reduced and even though this was not an objective of our study, we can note that only one significant correlation was obtained, which indicated that the greater the control of response inhibition, the better the updating ability. Therefore, our data do not allow us to confirm the existence, at a global level, of a relationship between all the EFs studied (see **Table 3**). However, Miyake et al. (2000), who studied the relationship between three of the EFs analyzed in this study (attentional shifting, updating, and inhibition), found moderate correlations between them, confirming that although they are three distinct entities, they were not entirely independent of each other, but rather that they shared common underlying characteristics.

In summary, in this study carried out on children with ADHD, deficits in EFs such as updating and attentional shifting, as well as in verbal span were found. It is noteworthy that all the affected functions are especially related to WM, that is, with the ability to remember information over a short period of time and mentally use this information to learn, understand, and reason. Regarding the divided attention, paradoxically, ADHD participants were less affected than the TD when a secondary task was added to the primary task, thus having to divide their attention and cognitive resources between them. This finding may highlight the importance of the motivational variables in children with ADHD, making it possible for them to strive more and get less distracted in tasks they consider as a challenge.

Finally, it is important to note the importance of evaluating the different EFs within the clinical setting in the cases of ADHD, in order not to use the results as a diagnostic decision criteria, as these deficits do not seem to be specific to this disorder, but rather to use them with the objective of obtaining valuable information regarding the cognitive functioning of each child. Furthermore, during clinical interventions, these data will aid in the creation of an intervention plan for each child according to the deficits found and will orientate the professional in relation to the prognosis, as greater difficulties in EFs entail worse performance at a relationship, educational and behavioral level in the future (see Duff and Sulla, 2015). Thus, it is essential that the cognitive difficulties found are taken into account, training such EFs and supplying tools to compensate problems. It is also necessary to adapt school and everyday tasks, obtaining optimum levels of difficulty so that their performance becomes motivating. More precisely, among the specific benefits for education, the results of the present study suggest, on the one hand, that in order to decrease the updating difficulties, tasks need to be broken down into smaller steps and each step must be reinforced. On the other hand, structurizing tasks into shorter times may help children with ADHD to concentrate better and finish tasks successfully, which will reflect onto their academic performance.

## Limitations

In relation to this present study, the following limitations are noteworthy. Firstly, ADHD is a clinically heterogeneous disorder with a high rate of comorbid conditions, thus making it extremely difficult to completely control the comorbidity of a representative sample of children with ADHD. It is relevant to note the importance of controlling comorbid emotional problems and behavior problems of children with ADHD, which were not satisfactorily controlled. In the present study, ADHD was the main diagnosis, without any other diagnosis, although comorbid emotional or behavioral symptomatology may exist in some cases, without constituting a disorder in themselves. Another possible defining variable is the predominant subtype of ADHD that participants suffer, which led this study to analyze the results globally, because in the sample used, the number of participants in each subtype was asymmetrical, hence making more detailed analyses impossible. As for the generalization of this study, the sample size was small and the ADHD group was limited by the low number of females among the participants. Thus, the result of this study may be more applicable to the male population with ADHD, which is the population that most frequently suffers this disorder.

## Conclusion

Our study adds to this research field results in favor of the existence of alterations in the EFs of children with ADHD, although not all of them would be affected, with relevant differentiation in the specific performance of each EF (divided attention, updating, attentional shifting, and inhibition). We can conclude that our study supported the hypothesis that EF deficits are an important component of the ADHD neuropsychology, although they are not sufficient to fully explain its symptomatology.

This research, with its comprehensive review of previous literature and contradictory results in some of the cognitive functions analyzed, highlights the need to ratify in ADHD the prior results for each of the four EFs studied. Furthermore, this study provides a combination of clinical reality and experimental rigor on a sample of children with ADHD, recently suggested by Snyder et al. (2015), who underlined the need for greater exactitude when comparing results and the need to include experimental controls when implementing tasks.

## Author Contributions

Conceived and designed the tasks: ME and MC; Performed the experiment: SD; Analyzed the data: ME, MC, and SD. Interpretation of the data: ME, MC, and SD. Drafted the paper: ME, MC, and SD. Contribution to the redaction: ME, MC, and SD.

## Conflict of Interest Statement

The authors declare that the research was conducted in the absence of any commercial or financial relationships that could be construed as a potential conflict of interest.
